# Supplementary material for: Spatial control of lipid droplet proteins by the ERAD ubiquitin ligase Doa10
Source: EMBO J. 2016 Jun 29;35(15):1644–55. doi: 10.15252/embj.201593106 (PMC4969576; doi:10.15252/embj.201593106)
Supplement: Supplementary file 1 — Expanded View Figures PDF [file EMBJ-35-1644-s001.pdf]

Expanded View Figures

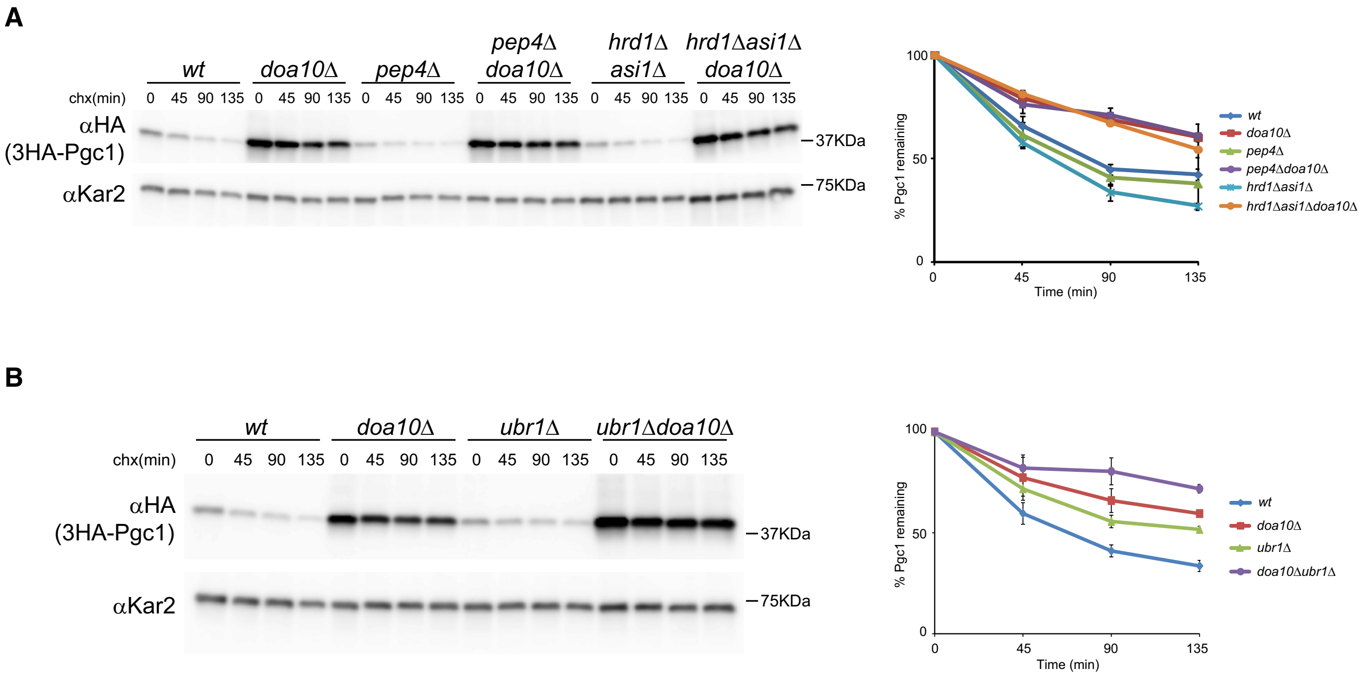

Figure EV1. Pgc1 degradation is independent of the vacuolar protease Pep4 but requires Doa10 and the cytoplasmic ubiquitin ligase Ubr1.

A, B The degradation of 3HA-Pgc1 was analyzed in cells with the indicated genotype as described in Fig 1A. The graphs show the average of two independent experiments; error bars represent the standard deviation.

© 2016 The Authors

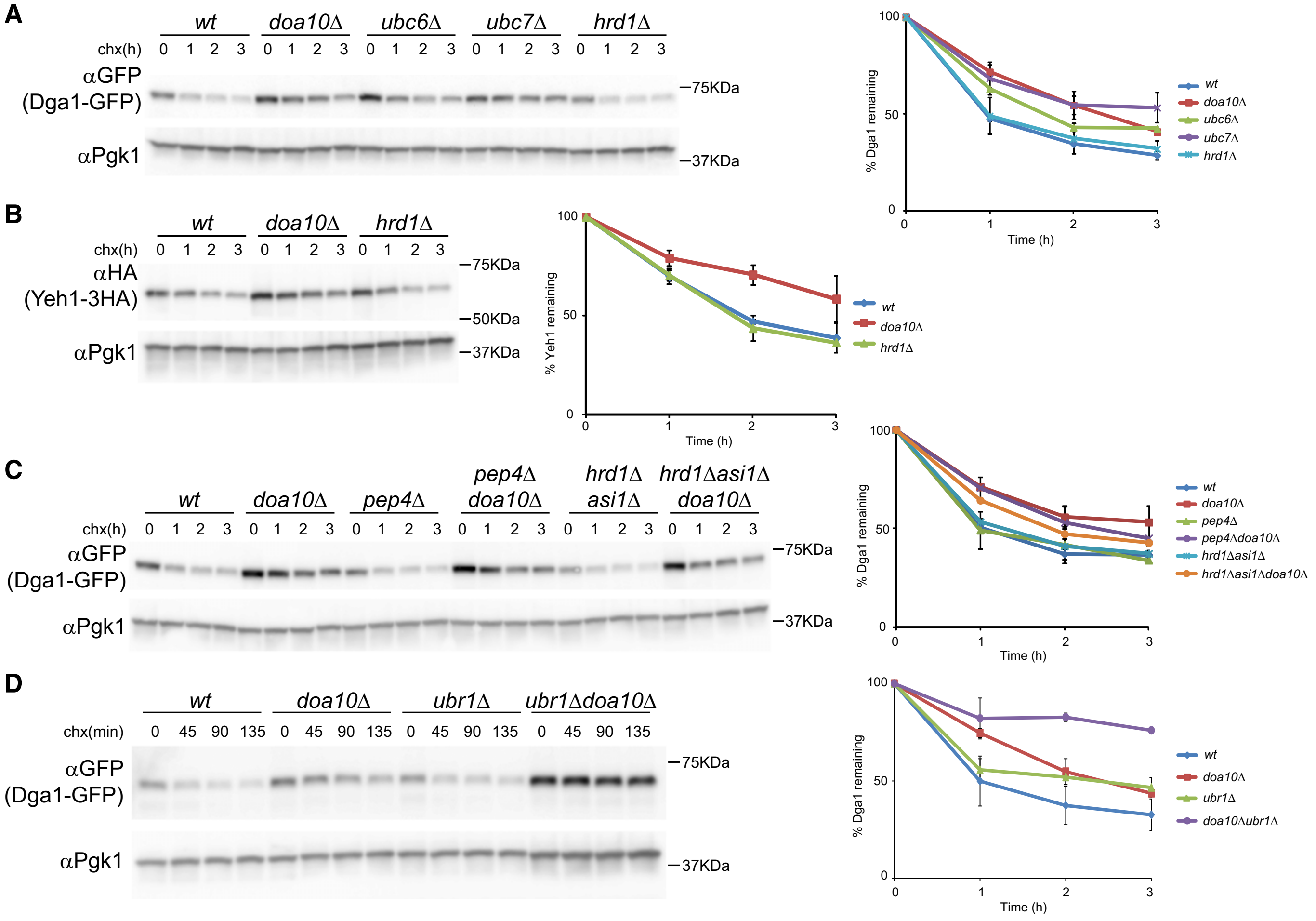

Figure EV2. The LD proteins Dga1 and Yeh1 are degraded by the ERAD ubiquitin ligase Doa10.

A The degradation of Dga1-GFP was analyzed in cells with the indicated genotype as described in Fig 1A. A plasmid-borne GFP-Dga1 expressed from the constitutive *ADH1* promoter was used. The graph shows the average of two independent experiments; error bars represent the standard deviation.

B The degradation of Yeh1-3HA was analyzed in cells with the indicated genotype as described in Fig 1A. A plasmid-borne Yeh1-3HA expressed from the endogenous promoter was used. The graph shows the average of two independent experiments; error bars represent the standard deviation.

C, D The degradation of Dga1-GFP was analyzed in cells with the indicated genotype as described in Fig 1A. The graphs show the average of two independent experiments; error bars represent the standard deviation.

© 2016 The Authors

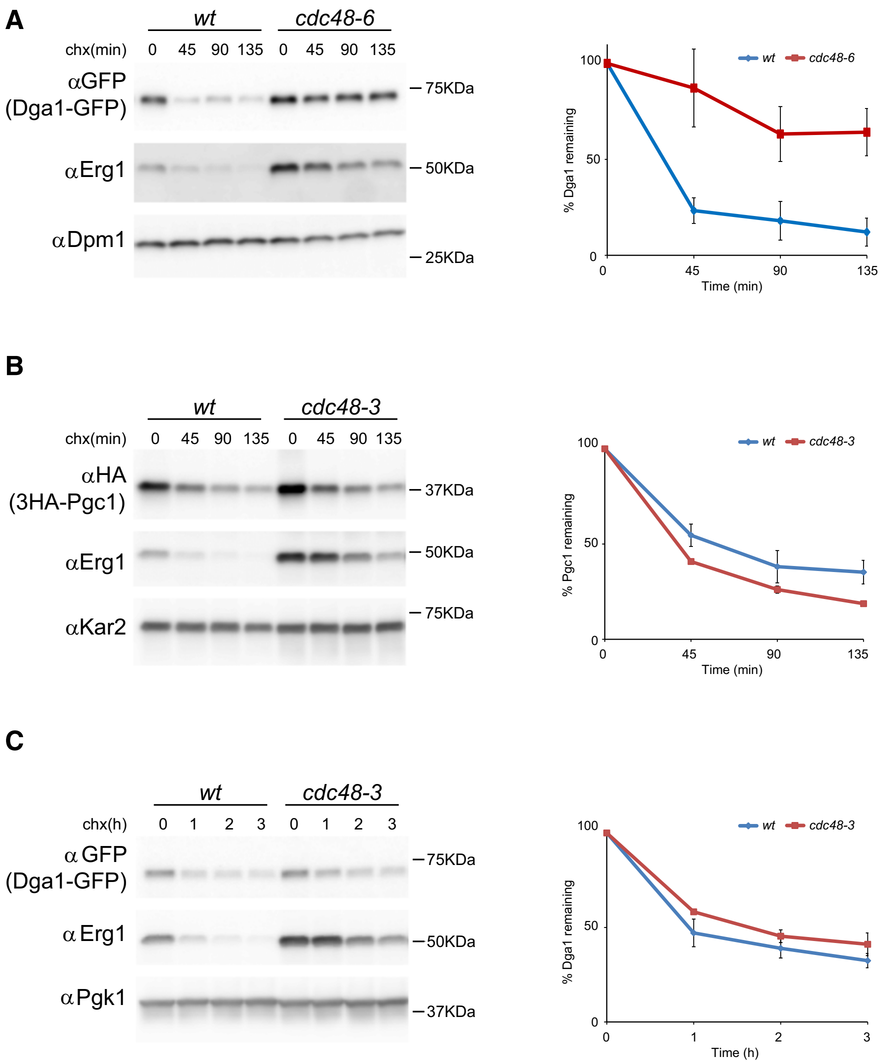

Figure EV3. Degradation of Pgc1 and Dga1 in *cdc48* mutant cells.

A The degradation of Dga1-GFP in temperature-sensitive *cdc48-6* mutant cells was analyzed as in Fig 1C. The graph shows the average of three independent experiments; error bars represent the standard deviation.

B, C The degradation of 3HA-Pgc1 and Dga1-GFP in temperature-sensitive *cdc48-3* mutant cells was analyzed as described in Fig 1C. The graphs show the average of two independent experiments; error bars represent the standard deviation.

© 2016 The Authors

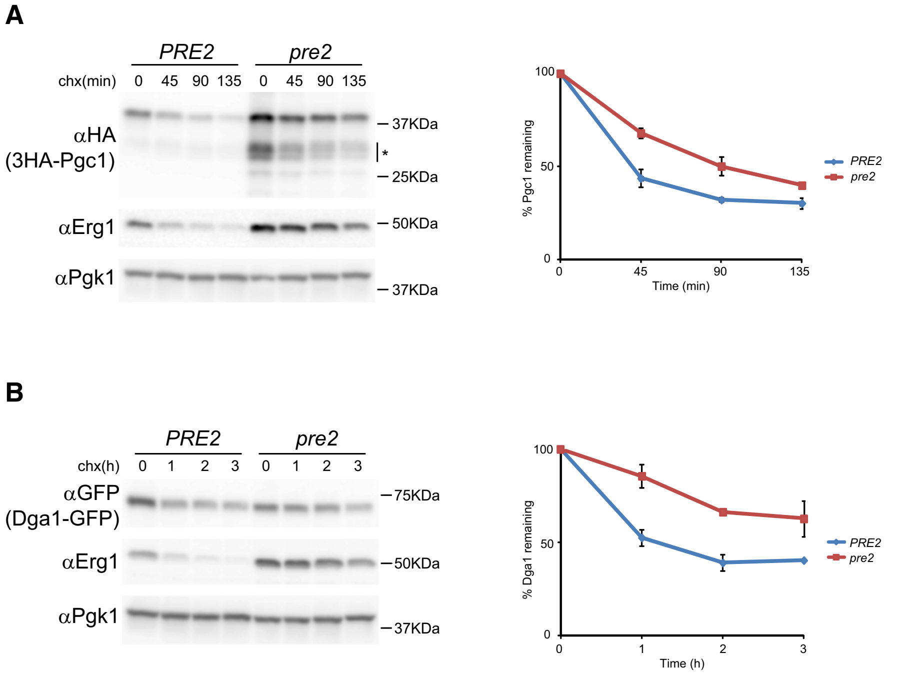

Figure EV4. Pgc1 and Dga1 are degraded by the proteasome.

A The degradation of 3HA-Pgc1 was analyzed in proteasome-deficient (*pre2*) and wt control (*PRE2*) cells as described in Fig 1A. Asterisk indicates degradation products accumulating in proteasome-deficient cells. The graph shows the average of two independent experiments; error bars represent the standard deviation.

B The degradation of Dga1-GFP was analyzed in proteasome-deficient (*pre2*) and wt control (*PRE2*) cells as in Fig 1A. The graph shows the average of two independent experiments; error bars represent the standard deviation.

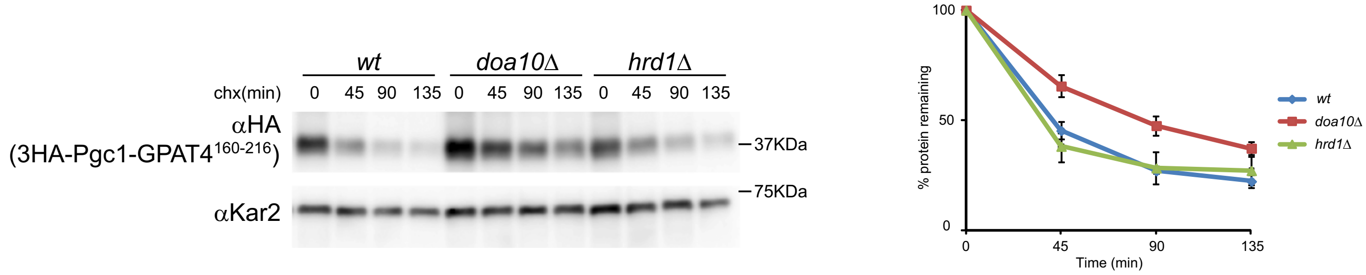

Figure EV5. The chimeric protein 3HA-Pgc1-GPAT$^{160–216}$ is a Doa10 substrate.
The degradation of 3HA-Pgc1-GPAT$^{160–216}$ was analyzed in cells with the indicated genotype as in Fig 1A. The graph shows the average of three independent experiments; error bars represent the standard deviation.

 © 2016 The Authors
