# Supplementary material for: Spatial control of lipid droplet proteins by the ERAD ubiquitin ligase Doa10
Source: EMBO J. 2016 Jun 29;35(15):1644–55. doi: 10.15252/embj.201593106 (PMC4969576; doi:10.15252/embj.201593106)
Supplement: Supplementary file 2 — Table EV1 [file EMBJ-35-1644-s008.docx]

**Table EV1. Yeast strains used in this study**

| **Strain** | **Genotype** |
| --- | --- |
| yPC4314 | *Matα ura3∆0 his3∆1 leu2∆0 lys2∆0 SEC63-mCHERRY* |
| yPC5686 | *Mata ura3-52 his3∆200 leu2∆1 trp1∆63*  *< PRC1pr-Vma12-NDC10C’ ^902-956^-3HA , URA, CEN >* |
| yPC5688 | *Mata ura3-52 his3∆200 leu2∆1 trp1∆63 doa10::HygB*  *< PRC1pr-Vma12-NDC10C’ ^902-956^-3HA , URA, CEN >* |
| yPC6800 | *Mata ura3∆0 his3∆1 leu2∆0 met15∆0 NAT-TEF-3HA-PGC1* |
| yPC6802 | *Mata NAT-TEF-3HA-PGC1 doa10::HygB* |
| yPC6803 | *Mata NAT-ADH1-GFP-PGC1doa10::HygB* |
| yPC6834 | *Mat? NAT-ADH1-GFP-PGC1* |
| yPC7014 | *Mata ura3∆0 his3∆1 leu2∆0 met15∆0 <3HA-PGC1, CEN, LEU>* |
| yPC7015 | *Mata ura3∆0 his3∆1 leu2∆0 met15∆0 doa10::HygB <3HA-PGC1, CEN, LEU>* |
| yPC7016 | *Mata ura3∆0 his3∆1 leu2∆0 ubc6::KANR <3HA-PGC1, CEN, LEU>* |
| yPC7017 | *Matα ura3∆0 his3∆1 leu2∆0 lys2∆0 ubc7::KANR <3HA-PGC1, CEN, LEU>* |
| yPC7018 | *Mata ura3∆0 his3∆1 leu2∆0 met15∆0 hrd1::HygB <3HA-PGC1, CEN, LEU>* |
| yPC7019 | *Mata are1::KANR are2::HYGB lro1::KANR dga1::NAT <3HA-PGC1, CEN, LEU>* |
| yPC7074 | *Mat? are1::KANR are2::HYGB lro1::KANR dga1::NAT doa10::HIS*  *<3HA-PGC1, CEN, LEU>* |
| yPC7249 | *Mata ura3∆0 his3∆1 leu2∆0 met15∆0 NAT-ADH1-DGA1-GFP-HIS2* |
| yPC7589 | *Mata are1::KANR are2::HYGB lro1::KANR dga1::NAT <GFP-PGC1, CEN, LEU>*  *<SEC63-mCherry, CEN, URA>* |
| yPC7590 | *Mat? are1::KANR are2::HYGB lro1::KANR dga1::NAT doa10::HIS*  *<GFP-PGC1, CEN, LEU> <SEC63-mCherry, CEN, URA>* |
| yPC8115 | *Mat? are1::KANR are2::HYGB lro1::KANR dga1::NAT hrd1::HIS <3HA-PGC1, CEN, LEU>* |
| yPC8150 | *Mata ura3∆0 his3∆1 leu2∆0 met15∆0 <3HA-PGC1^Bos1MA^, CEN, LEU>* |
| yPC8151 | *Mata ura3∆0 his3∆1 leu2∆0 met15∆0 doa10::HygB <3HA-PGC1^Bos1MA^, CEN, LEU>* |
| yPC8152 | *Mata ura3∆0 his3∆1 leu2∆0 met15∆0 <3HA-PGC1^Scs2MA^, CEN, LEU>* |
| yPC8153 | *Mata ura3∆0 his3∆1 leu2∆0 met15∆0 doa10::HygB <3HA-PGC1^Scs2MA^, CEN, LEU>* |
| yPC8336 | *Mata ura3∆0 his3∆1 leu2∆0 met15∆0 doa10::HygB <3HA-GFP-PGC1^275-321^, CEN, LEU> <SEC63-mCherry, CEN, URA>* |
| yPC8412 | *Mata ura3-52 his3∆200 leu2∆1 trp1∆63 cdc48-3 <3HA-PGC1, CEN, LEU>* |
| yPC8413 | *Mata ura3-52 leu2∆1 trp1∆63 npl4-1 <3HA-PGC1, CEN, LEU>* |
| yPC8620 | *Mata ura3∆0 his3∆1 leu2∆0 met15∆0 <ADH1-DGA1-GFP, CEN, LEU>* |
| yPC8621 | *Mata ura3∆0 his3∆1 leu2∆0 met15∆0 doa10::HygB <ADH1-DGA1-GFP, CEN, LEU>* |
| yPC8928 | *Mata ura3∆0 his3∆1 leu2∆0 met15∆0 NAT-GAL1-GFP-PGC1* |
| yPC8934 | *Mata ura3∆0 his3∆1 leu2∆0 ubc6::KANR <ADH1-DGA1-GFP, CEN, LEU>* |
| yPC8935 | *Matα ura3∆0 his3∆1 leu2∆0 lys2∆0 ubc7::KANR <ADH1-DGA1-GFP, CEN, LEU>* |
| yPC8936 | *Matα hrd1::HIS <ADH1-DGA1-GFP, CEN, LEU>* |
| yPC8937 | *Mata ura3-52 his3∆200 leu2∆1 trp1∆63 cdc48-3 <ADH1-DGA1-GFP, CEN, LEU>* |
| yPC8938 | *Mata ura3-52 leu2∆1 trp1∆63 npl4-1 <ADH1-DGA1-GFP, CEN, LEU>* |
| yPC8939 | *Mat? doa10::KANR NAT-GAL1-GFP-PGC1* |
| yPC8941 | *Matα pre2 <3HA-PGC1, CEN, LEU>* |
| yPC8942 | *Mat? PRE2 <3HA-PGC1, CEN, LEU>* |
| yPC8943 | *Matα pre2 <ADH1-DGA1-GFP, CEN, LEU>* |
| yPC8944 | *Mat? PRE2 <ADH1-DGA1-GFP, CEN, LEU>* |
| yPC8973 | *Mata ura3∆0 his3∆1 leu2∆0 met15∆0 <GFP- PGC1^Scs2MA^, CEN, LEU>*  *<SEC63-mCherry, CEN, URA>* |
| yPC8974 | *Mata ura3∆0 his3∆1 leu2∆0 met15∆0 doa10::HygB <GFP-PGC1^Scs2MA^, CEN, LEU> <SEC63-mCherry, CEN, URA>* |
| yPC8975 | *Mata ura3∆0 his3∆1 leu2∆0 met15∆0 <GFP-PGC1^Bos1MA^, CEN, LEU>*  *<SEC63-mCherry, CEN, URA>* |
| yPC8976 | *Mata ura3∆0 his3∆1 leu2∆0 met15∆0 doa10::HygB <GFP-PGC1^Bos1MA^, CEN, LEU> <SEC63-mCherry, CEN, URA>* |
| yPC8977 | *Mata ura3∆0 his3∆1 leu2∆0 met15∆0 <3HA-GFP-PGC1^275-321^, CEN, LEU>* |
| yPC8978 | *Mata ura3∆0 his3∆1 leu2∆0 met15∆0 doa10::HygB <3HA-GFP-PGC1^275-321^, CEN, LEU>* |
| yPC8980 | *Mat? pep4::URA3 <3HA-PGC1, CEN, LEU>* |
| yPC8981 | *Mat? pep4::URA3 doa10::KANR <3HA-PGC1, CEN, LEU>* |
| yPC8982 | *Mat? pep4::URA3 <ADH1-DGA1-GFP, CEN, LEU>* |
| yPC8983 | *Mat? pep4::URA3 doa10::KANR <ADH1-DGA1-GFP, CEN, LEU>* |
| yPC8984 | *Mata ura3∆0 his3∆1 leu2∆0 met15∆0 <3HA-PGC1-GPAT4^160-216^, CEN, LEU>* |
| yPC8985 | *Mata ura3∆0 his3∆1 leu2∆0 met15∆0 doa10::HygB*  *<3HA-PGC1-GPAT4^160-216^, CEN, LEU>* |
| yPC8986 | *Matα hrd1::HIS <3HA-PGC1-GPAT4^160-216^, CEN, LEU>* |
| yPC8987 | *Mata are1::KANR are2::HYGB lro1::KANR dga1::NAT <3HA-PGC1-GPAT4^160-216^, CEN, LEU>* |
| yPC8988 | *Mat? are1::KANR are2::HYGB lro1::KANR dga1::NAT doa10::HIS <3HA-PGC1-GPAT4^160-216^, CEN, LEU>* |
| yPC8989 | *Mat? are1::KANR are2::HYGB lro1::KANR dga1::NAT hrd1::HIS <3HA-PGC1-GPAT4^160-216^, CEN, LEU>* |
| yPC8992 | *Mat? are1::KANR are2::HYGB lro1::KANR dga1::NAT doa10::HIS <GFP-PGC1-GPAT4^160-216^, CEN, LEU> <SEC63-mCherry, CEN, URA>* |
| yPC9005 | *Mat? his3∆1 hrd1::KANR asi1::NATR <3HA-PGC1, CEN, LEU>* |
| yPC9006 | *Mat? his3∆1 hrd1::KANR asi1::NATR <ADH1-DGA1-GFP, CEN, LEU>* |
| yPC9007 | *Mata doa10::HIS hrd1::KANR asi1::NATR <3HA-PGC1, CEN, LEU>* |
| yPC9008 | *Mata doa10::HIS hrd1::KANR asi1::NATR <ADH1-DGA1-GFP, CEN, LEU>* |
| yPC9214 | *Mata ura3∆0 his3∆1 leu2∆0 met15∆0 YEH1-3HA* |
| yPC9231 | *Mata ura3∆0 his3∆1 leu2∆0 met15∆0 <YEH1-3HA, CEN, URA>* |
| yPC9232 | *Mata ura3∆0 his3∆1 leu2∆0 met15∆0 doa10::HygB <YEH1-3HA, CEN, URA>* |
| yPC9233 | *Matα hrd1::HIS <YEH1-3HA, CEN, URA>* |
| yPC9417 | *Mat? ura3∆0 his3∆1 leu2∆0 met15∆0 are1::KANR are2::HYGB lro1::HIS KANR-GAL1-DGA1 Erg6-mCherry-URA <ADH1-GFP-PGC1, CEN, LEU>* |
| yPC9418 | *Mata ura3∆0 his3∆1 leu2∆0 met15∆0 <ADH1-GFP-PGC1-GPAT4^160-216^, CEN, LEU> <SEC63-mCherry, CEN, URA>* |
| yPC9419 | *Mata ura3∆0 his3∆1 leu2∆0 met15∆0 doa10::HygB*  *<ADH1-GFP-PGC1-GPAT4^160-216^, CEN, LEU> <SEC63-mCherry, CEN, URA>* |
| yPC9420 | *Mata ura3∆0 his3∆1 leu2∆0 met15∆0 doa10::HygB NAT-ADH1-GFP-PGC1*  *<SEC63-mCherry, CEN, URA>* |
| yPC9421 | *Mat? NAT-ADH1-GFP-PGC1 <SEC63-mCherry, CEN, URA>* |
| yPC9449 | *Mata ura3-52 his3∆200 leu2∆1 trp1∆63 <3HA-PGC1, CEN, LEU>*  *<MYC-UBIQUITIN, 2µm, TRP>* |
| yPC9450 | *Mata ura3-52 his3∆200 leu2∆1 trp1∆63 doa10::HygB <3HA-PGC1, CEN, LEU>*  *<MYC-UBIQUITIN, 2µm, TRP>* |
| yPC9509 | *Mata ura3∆0 his3∆1 leu2∆0 met15∆0 ubr1::KANR <3HA-PGC1, CEN, LEU>* |
| yPC9510 | *Mata ura3∆0 his3∆1 leu2∆0 met15∆0 ubr1::KANR <ADH1-DGA1-GFP, CEN, LEU>* |
| yPC9511 | *Mat? ubr1::KANR doa10::HIS <3HA-PGC1, CEN, LEU>* |
| yPC9512 | *Mat? ubr1::KANR doa10::HIS <ADH1-DGA1-GFP, CEN, LEU>* |
| yPC9595 | *Mata ura3∆0 his3∆1 leu2∆0 met15∆0 ubr1::KANR <3HA-PGC1-GPAT4^160-216^, CEN, LEU>* |
| yPC9596 | *Mat? ubr1::KANR doa10::HIS <3HA-PGC1-GPAT4^160-216^, CEN, LEU>* |
| yPC9551 | *Mat? ubr1::KANR doa10::HIS <ADH1-GFP-PGC1-GPAT4^160-216^, CEN, LEU>*  *<SEC63-mCherry, CEN, URA>* |
| yPC9821 | *his3∆200 leu2-3 2-112 lys2-801 ura3-52 trp1-1(am) <3HA-PGC1, CEN, LEU>* |
| yPC9823 | *cdc48-6 his3∆200 leu2-3 2-112 lys2-801 ura3-52 trp1-1(am) <3HA-PGC1, CEN, LEU>* |
| yPC9845 | *tgl3::KANR tgl4::NAT tgl5::HygB met15∆0, ura3∆0, leu2∆0, his3∆1 <3HA-PGC1, CEN, LEU>* |
| yPC10071 | *Mata ura3∆0 his3∆1 leu2∆0 met15∆0 are1::KANR are2::HYGB lro1::HIS KANR-GAL1-DGA1 doa10::URA3 <ADH1-tdEOS-PGC1, CEN, LEU>* |
| yPC10082 | *Mata ura3-52 his3∆200 leu2∆1 trp1∆63 cdc48-3 <3HA-PGC1, CEN, LEU>*  *<MYC-UBIQUITIN, 2µm, TRP>* |
| yPC10083 | *his3∆200, leu2-3, 2-112, lys2-801, ura3-52, trp1-1(am) <3HA-PGC1, CEN, LEU>*  *<MYC-UBIQUITIN, 2µm, TRP>* |
| yPC10084 | *cdc48-6 his3∆200, leu2-3, 2-112, lys2-801, ura3-52, trp1-1(am) <3HA-PGC1, CEN, LEU>*  *<MYC-UBIQUITIN, 2µm, TRP>* |
|  |  |
