# Supplementary material for: Spatial control of lipid droplet proteins by the ERAD ubiquitin ligase Doa10
Source: EMBO J. 2016 Jun 29;35(15):1644–55. doi: 10.15252/embj.201593106 (PMC4969576; doi:10.15252/embj.201593106)
Supplement: Supplementary file 3 — Table EV2 [file EMBJ-35-1644-s009.docx]

**Table EV2. Plasmids used in this study**

| **Name** | **Insert/gene** | **vector** | **Derived from/Reference** |
| --- | --- | --- | --- |
| pPC435 | *MYC-UBIQUITIN* | - | Formerly pUB141-myc-Ub |
| pPC882 | *Sec63-mCherry* | pRS416 | - |
| pPC926 | *VMA12-NDC10C’* | pRS316 | Furth *et al*., 2011 |
| pPC1040 | *3HA-PGC1* | pRS315 | - |
| pPC1051 | *GFP-PGC1* | pRS315 | - |
| pPC1145 | *ADH1-GFP-PGC1* | pRS315 | - |
| pPC1084 | *3HA-GFP-PGC1* | pRS315 | pPC1040/pPC1051 |
| pPC1168 | *3HA-PGC1^Scs2MA^* | pRS315 | pPC1040 |
| pPC1169 | *3HA-PGC1^Bos1MA^* | pRS315 | pPC1040 |
| pPC1170 | *3HA-GFP-PGC1^275-321^* | pRS315 | pPC1084 |
| pPC1196 | *ADH1pr-DGA1-GFP* | pRS415 | - |
| pPC1270 | *GFP-PGC1^Scs2MA^* | pRS315 | pPC1051 |
| pPC1271 | *GFP-PGC1^Bos1MA^* | pRS315 | pPC1051 |
| pPC1272 | *3HA-PGC1-GPAT4^160-216^* | pRS315 | pPC1040 |
| pPC1273 | *GFP-PGC1-GPAT4^160-216^* | pRS315 | pPC1051 |
| pPC1299 | *Yeh1-3HA* | pRS316 | - |
| pPC1305 | *ADH1pr- GFP-PGC1-GPAT4^160-216^* | pRS315 | pPC1145/pPC1273 |
| pPC1371 | *ADH1pr- tdEOS-PGC1* | pRS315 | pPC1145 |
