# Supplementary material for: Spatial control of lipid droplet proteins by the ERAD ubiquitin ligase Doa10
Source: EMBO J. 2016 Jun 29;35(15):1644–55. doi: 10.15252/embj.201593106 (PMC4969576; doi:10.15252/embj.201593106)
Supplement: Supplementary file 4 — Table EV3 [file EMBJ-35-1644-s010.docx]

| **Number** | **Name** | **For plasmid** | **Sequence** |
| --- | --- | --- | --- |
| 185 | Yos9R5 | pPC1196/ 1299/882 | CTATTGTACTCGAGCGAGGCAAGCTAAACAGATC |
| 721 | Sec63F XbaI | pPC882 | cgatcGCGCTCTAGAGATAGAACCTTAGGGCATATTTTC |
| 1515 | Pgc1-F2 | pPC1040/ 1051 | ccagtgtccaGAGCTCCTAAGTACCCAACAGAGGTT |
| 1516 | Pgc1pr-3HA FusionRv | pPC1040 | GAACATCGTATGGGTAACCCATCCTCGTGTCCTTGTTGTTATC |
| 1517 | Pgc1pr-3HA FusionFw | pPC1040 | GATAACAACAAGGACACGAGGATGGGTTACCCATACGATGTTC |
| 1518 | Pgc1-R1 | pPC1040/ 1051 | gcagttcagtcCTGCAGGGAGAATGGCATACACATATC |
| 1530 | Pgc1-Scs2TMRv | pPC1168/  1270 | CAAGATAAGGAGTGCAACCAATATGAATATACCCATCTTAATATGGACCCATTTGGA |
| 1531 | Pgc1-Scs2TMFw | pPC1168/  1270 | GCACTCCTTATCTTGGTTTTAGGATGGTTCTACAGATGATATCCATTCATTGACGT |
| 1752 | Pgc1pr-3HA-GFP PhusionRv | pPC1084 | TAATTCTTCACCTTTAGACATCGATGAATTCTCTGTCGGACC |
| 1753 | Pgc1pr-3HA-GFP PhusionFw | pPC1084 | GGTCCGACAGAGAATTCATCGATGTCTAAAGGTGAAGAATTA |
| 1779 | ADH1 F2 | pPC1196/  1305 | CTCAGAGGACAACACCTGTTG |
| 1820 | Pgc1-Scs2 QX3 | pPC1168/  1270 | GTTGCGCCCAGTCTGGCATCTGTAATGGGTATATTCATATTG |
| 1821 | Pgc1-Bos1TMFw | pPC1169/  1271 | TCTCTTGATCATAGGTATTTATTATGTGTTGAAATGGTTAAGATGATATCCATTCATTGACGTG |
| 1822 | Pgc1-Bos1TMRv2 | pPC1169/  1271 | AAATACCTATGATCAAGAGAATTAACGCGATCCAAAAGACTAGTACAGATGCCAGACTGGGCGCAAC |
| 1823 | GFP-Pgc1TMextended | pPC1084 | GCTGGTCCGACAGAGAATTCATCGGATGGGCTAAGATTCCATGC |
| 2091 | Pgc1pr-GFP FusionRv | pPC1051 | ATAATTCTTCACCTTTAGACATgcggccgcCCTCGTGTCCTTGTTGTTATC |
| 2092 | Pgc1pr-GFP FusionFw | pPC1051 | GATAACAACAAGGACACGAGGgcggccgcATGTCTAAAGGTGAAGAATTAT |
| 2148 | Yeh1-F3 | pPC1299 | CAATGTCGAAAGCGGCCGCCCAATATACATTCTCAAGTGTGC |
| 2065 | Pgc1-hAGPAT6 Rv2 | pPC1272/  1273/1305 | TTTTCCAGGAAATGAACTCGTAGTGTACAGATGCCAGACTGGGCGCAACA |
| 2066 | Pgc1-hAGPAT6 Fw2 | pPC1272/  1273/1305 | TGTTGCGCCCAGTCTGGCATCTGTACACTACGAGTTCATTTCCTGGAAAA |
| 2067 | Pgc1-hAGPAT6 Rv3 | pPC1272/  1273 | TATTCACGTCAATGAATGGATATCAATCCCGCTTGAAGGGCCCATCCTTC |
| 2068 | Pgc1-hAGPAT6 Fw3 | pPC1272/  1273 | GAAGGATGGGCCCTTCAAGCGGGATTGATATCCATTCATTGACGTGAATA |
| 2069 | Pgc1-R2 | pPC1272/  1273/1305 | gcagttcagtcTCTAGAGGAGAATGGCATACACATATC |

**Table EV3. Primers used in this study**
