# Supplementary figures and images for: Spatial control of lipid droplet proteins by the ERAD ubiquitin ligase Doa10
Source: EMBO J. 2016 Jun 29;35(15):1644–55. doi: 10.15252/embj.201593106 (PMC4969576; doi:10.15252/embj.201593106)

A

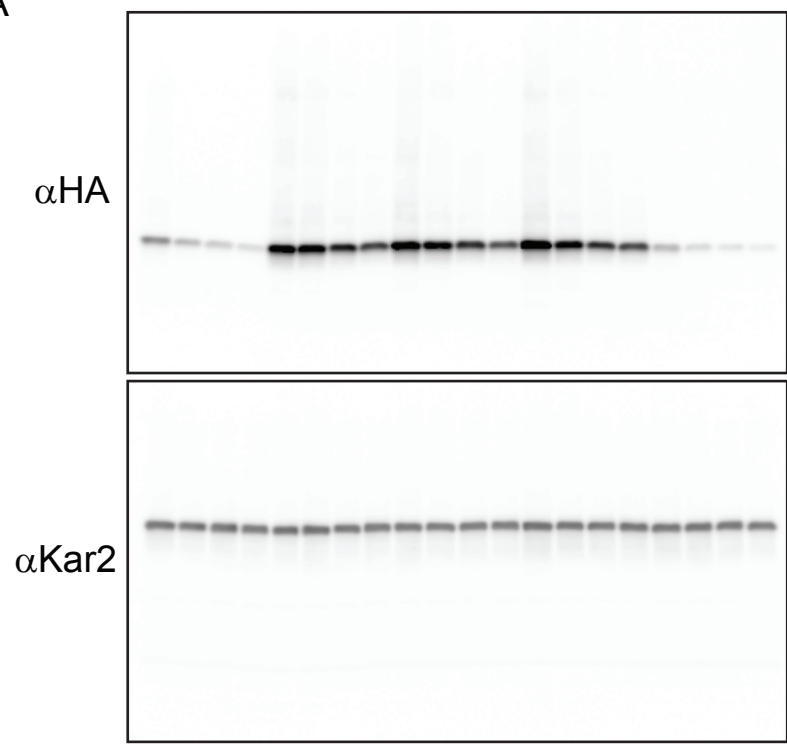

B

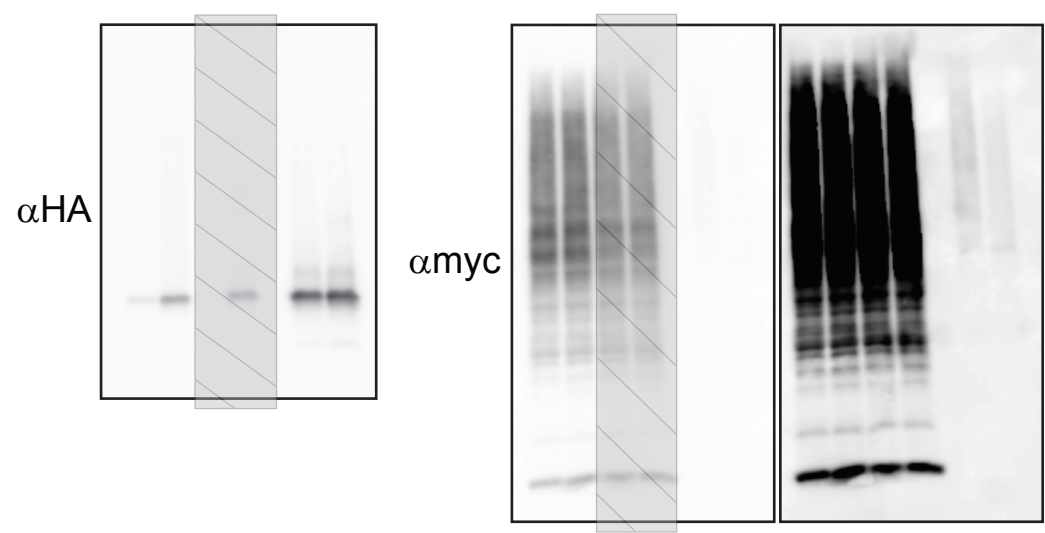

■ "IP unbound" lanes cut out in the final figure

C

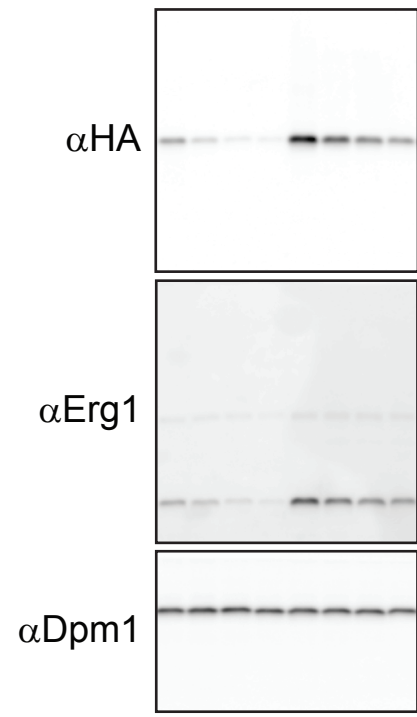

Supplement: Supplementary file 6 — Source Data for Figure 1 [file EMBJ-35-1644-s002.pdf]

C

$\alpha$ HA  
(left panel)

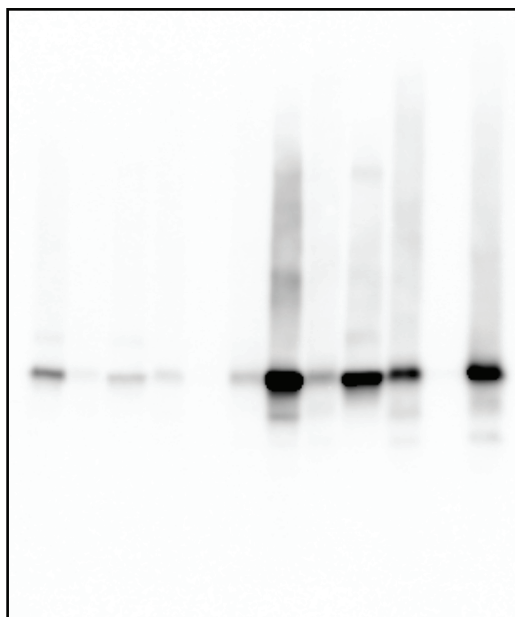

D

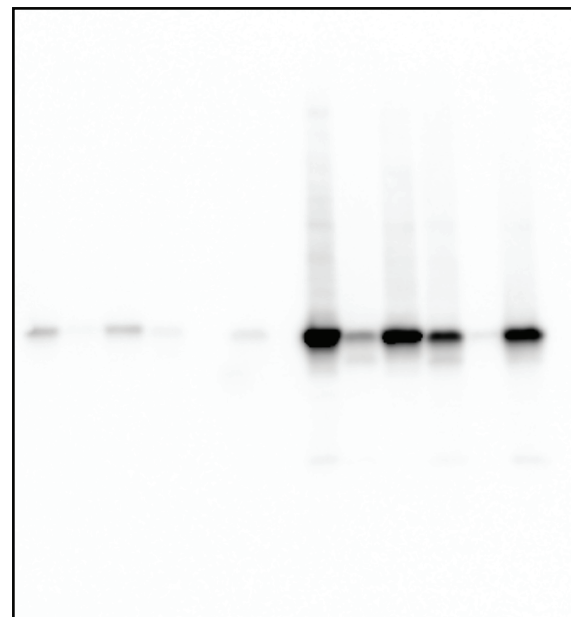

$\alpha$ HA  
(right panel)

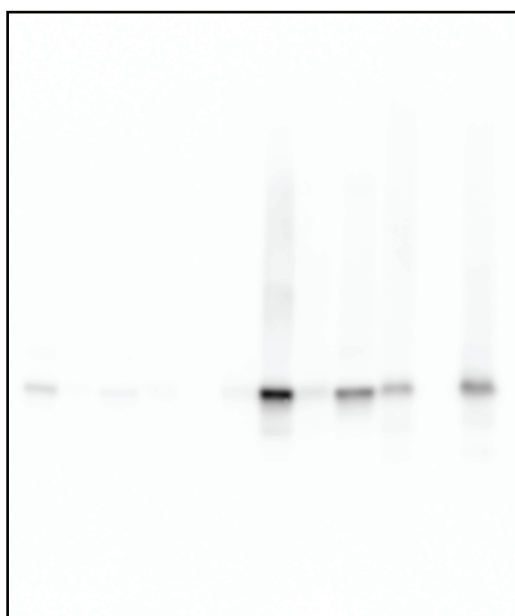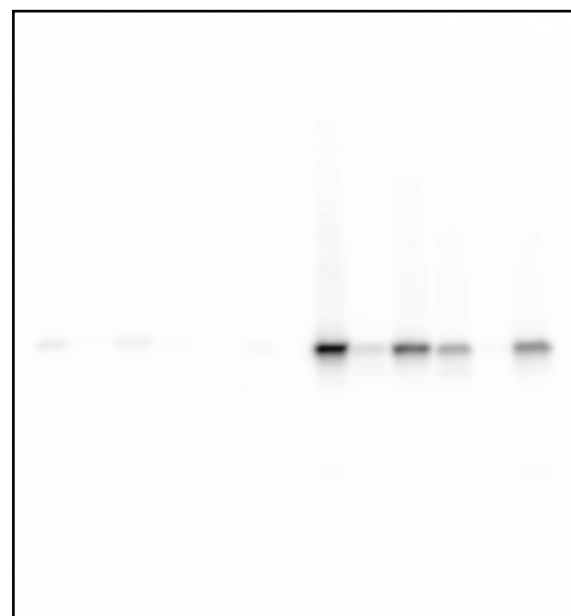

$\alpha$ Kar2

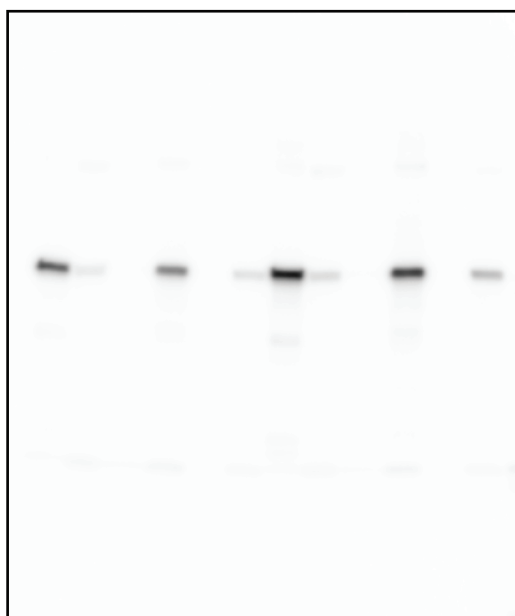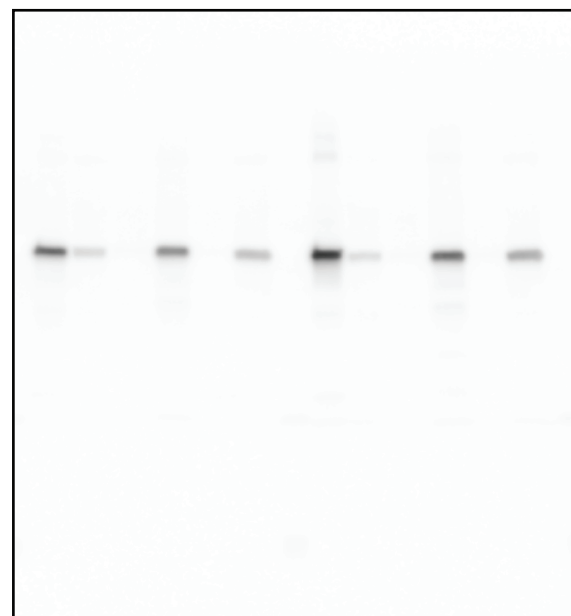

Supplement: Supplementary file 8 — Source Data for Figure 2 [file EMBJ-35-1644-s004.pdf]

A

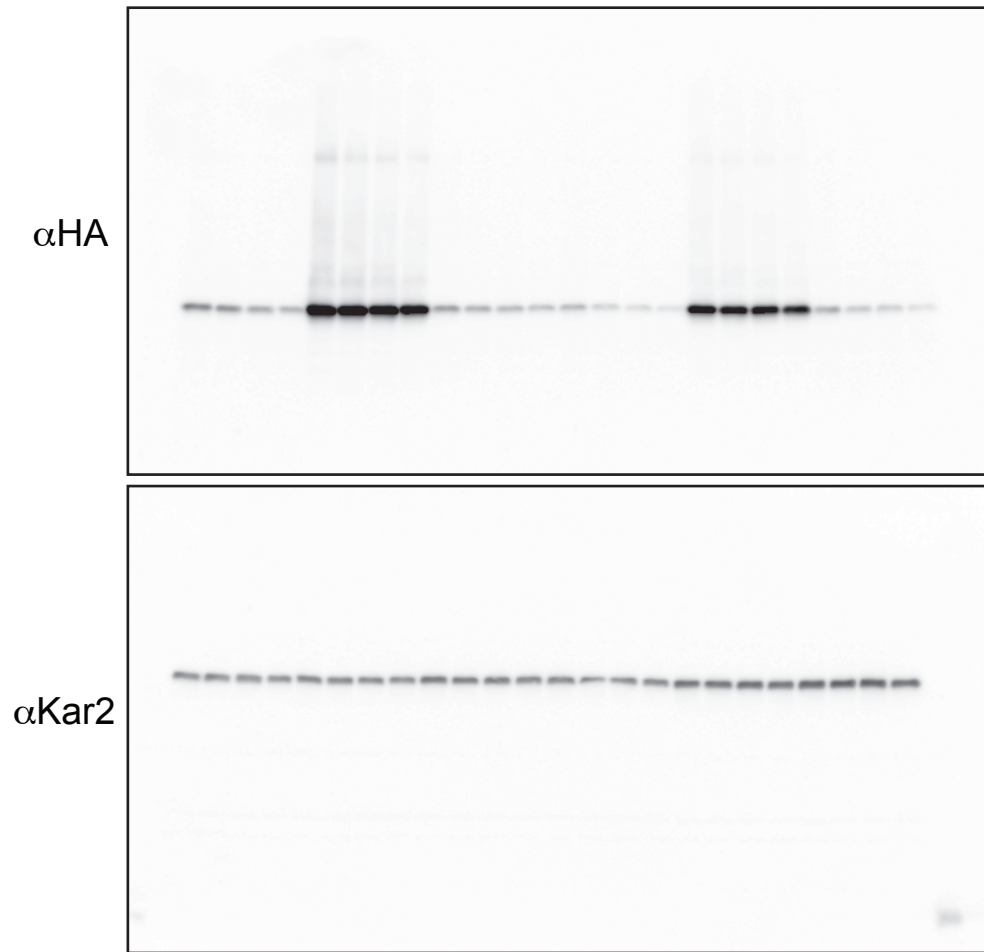

B

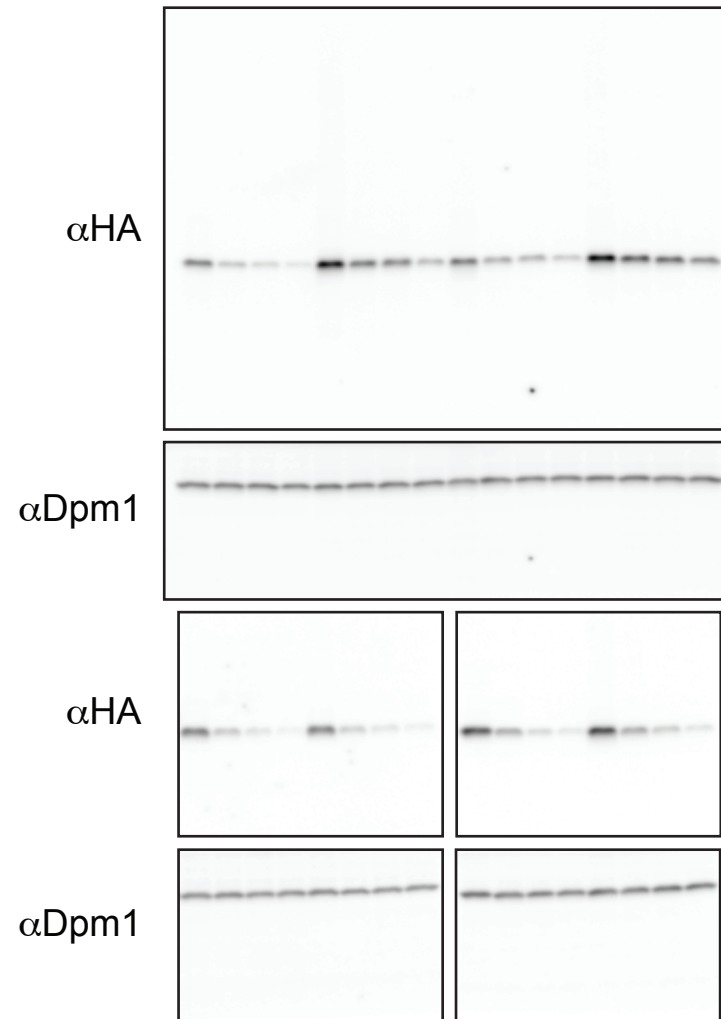

Supplement: Supplementary file 9 — Source Data for Figure 4 [file EMBJ-35-1644-s005.pdf]

B

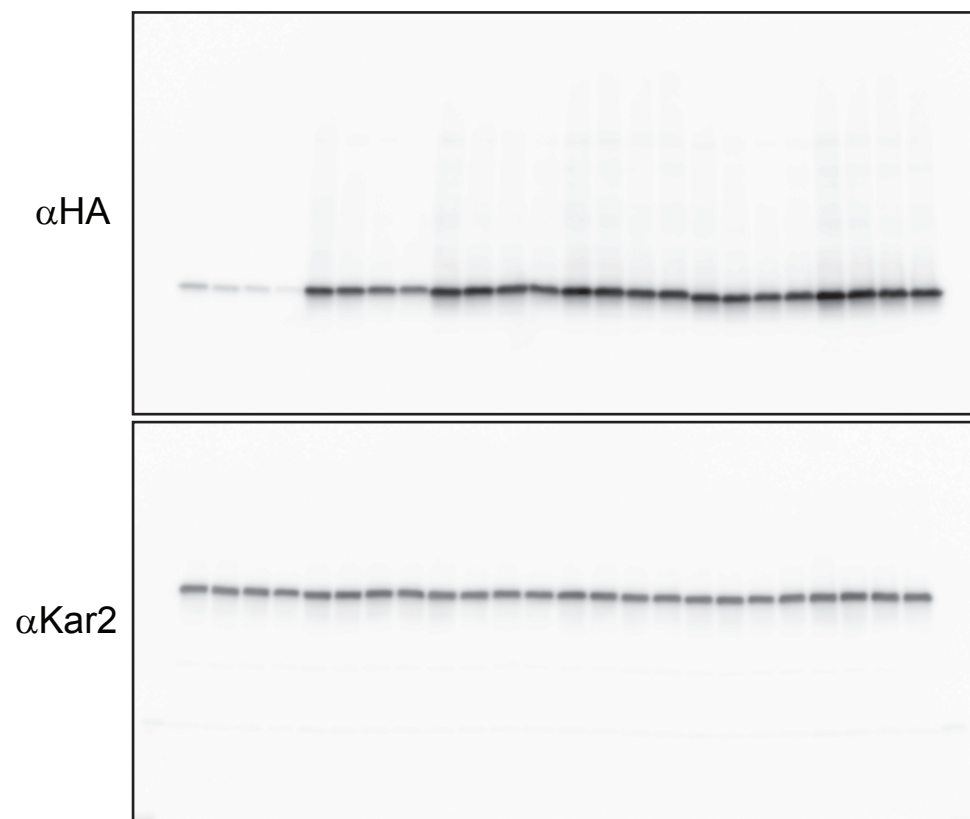

C

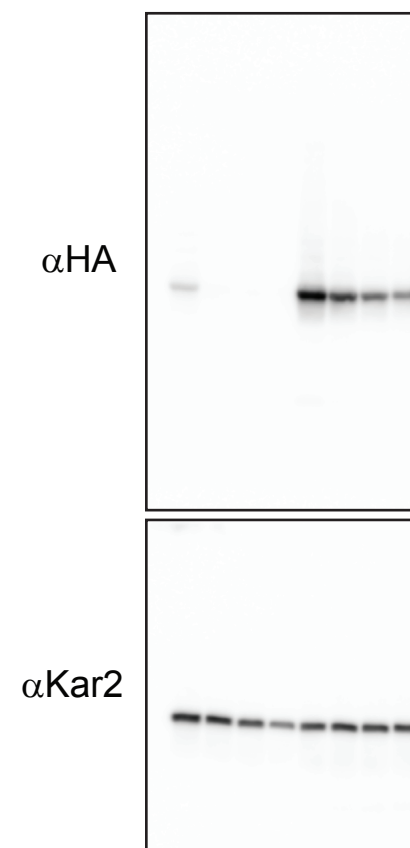

Supplement: Supplementary file 10 — Source Data for Figure 5 [file EMBJ-35-1644-s006.pdf]

A

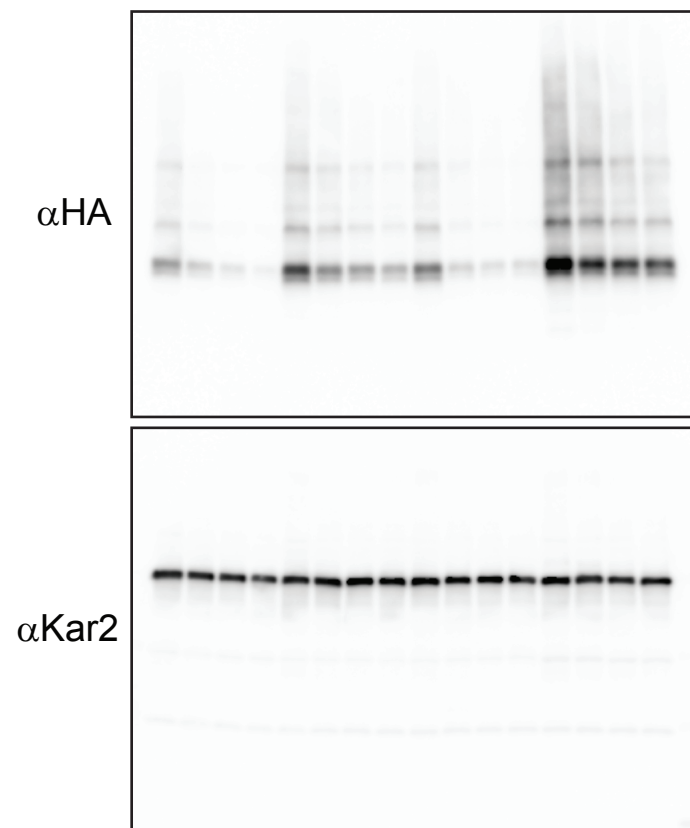

C

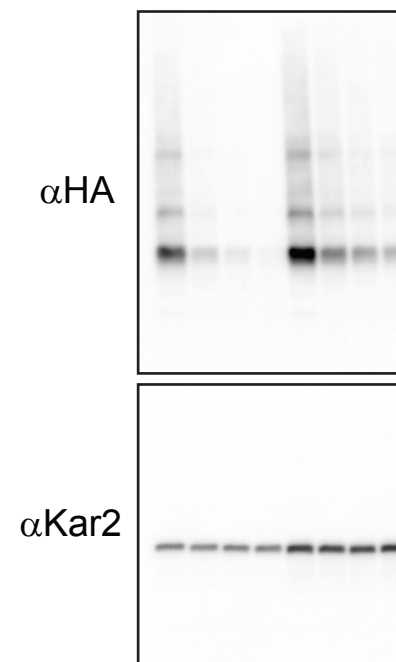

Supplement: Supplementary file 11 — Source Data for Figure 6 [file EMBJ-35-1644-s007.pdf]
