# Supplementary material for: Spatial control of lipid droplet proteins by the ERAD ubiquitin ligase Doa10
Source: EMBO J. 2016 Jun 29;35(15):1644–55. doi: 10.15252/embj.201593106 (PMC4969576; doi:10.15252/embj.201593106)
Supplement: Supplementary file 7 — Source Data for Figure 1D [file EMBJ-35-1644-s003.pdf]

D

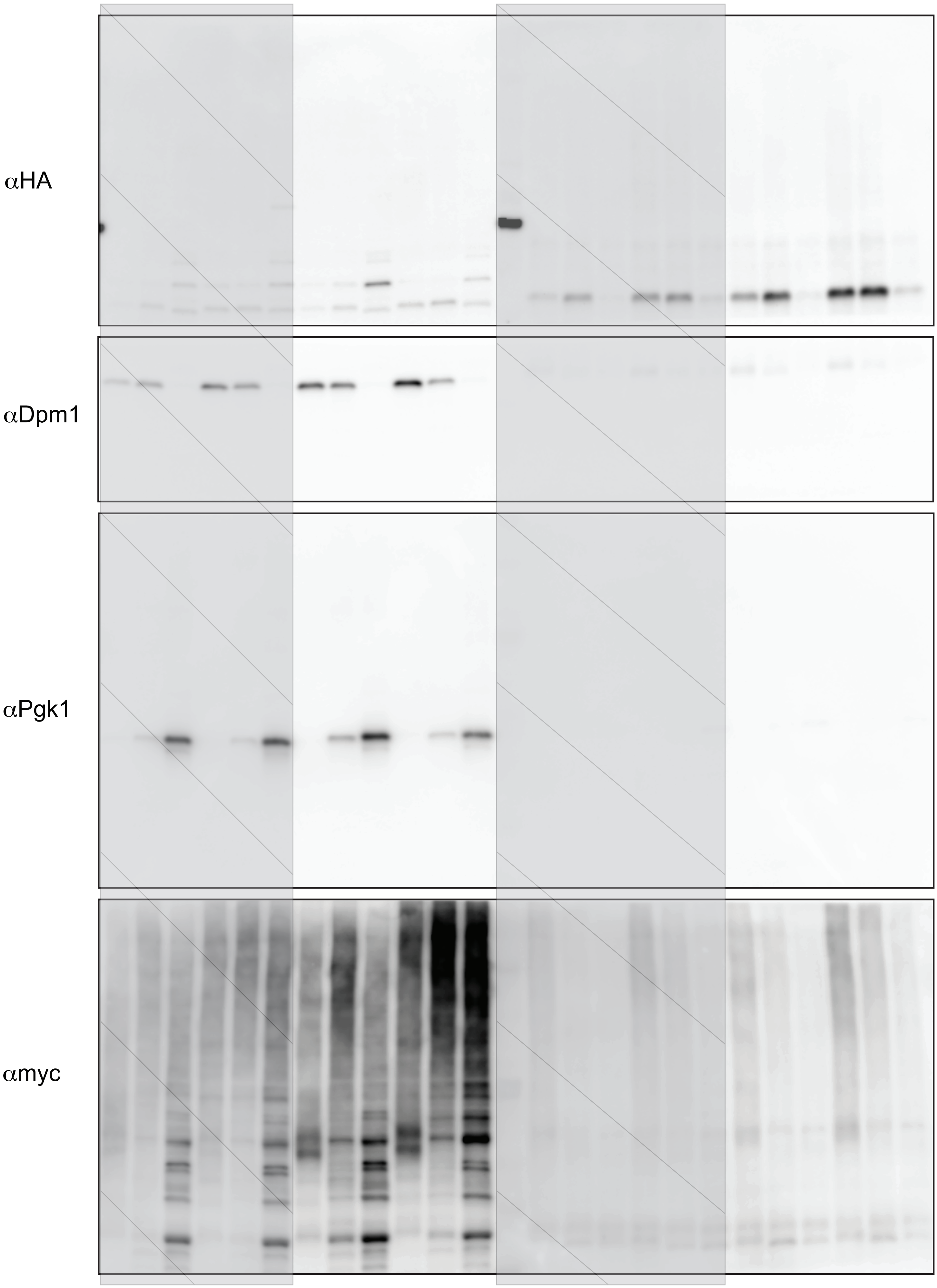

CDC48-3 and corresponding *wt* lanes cut out in the final figure
